# Effect of mouthwashes on the integrity of composite resin and resin modified glass ionomer: In vitro study

**DOI:** 10.4317/jced.55102

**Published:** 2019-02-01

**Authors:** Ana Armas-Vega, Paola Casanova-Obando, María-Fernanda Taboada-Alvear, Jonathan-Eduardo Aldas-Ramírez, Nadia Montero-Oleas, Andrés Viteri-García

**Affiliations:** 1 PhD. Center for Oral Health Research (CISO). Faculty of Health Sciences “Eugenio Espejo”. Universidad UTE; 2 DDS. Prosthodontist; 3 General Practice Dentist; 4 MD. MSc. Esp (c). Centro de Investigación en Salud Pública y Epidemiología Clínica (CISPEC). Faculty of Health Sciences “Eugenio Espejo”. Universidad UTE; 5 DDS. MSc. Centro de Investigación en Salud Pública y Epidemiología Clínica (CISPEC). Faculty of Health Sciences “Eugenio Espejo”. Universidad UTE

## Abstract

**Background:**

The constant search for an improved esthetic dental material has led investigators to realize that its performance depends on the conditions where the material is used. It has been probed that the contact with mouth rinses triggers alterations, reason why the aim of this study was to identify their possible effects of it on the integrity of nanohybrid composite resin and resin modified glass ionomer.

**Material and Methods:**

A total of 144 samples were manufactured with two nanohybrid composite resins and two resin modified glass ionomer restorative materials. The specimens were immersed in one of the three mouthwashes used in the study, for a total of 1092 minutes, with intervals of contact with artificial saliva. This strategy simulates three years of constant use of mouthwashes. The samples weight and surface roughness measurement was recorded with a precision scale and profilometer, at different stages: At the beginning of the study, after 546 minutes (simulating one and a half year), and after 1092 minutes (simulating three years).

**Results:**

The collected data on surface roughness and weight were submitted to the analysis of variance (ANOVA), with repeated measures of three factors. The results determined shifts in values in terms of weight and roughness in all the samples. The composite resin “Grandio” group was the one that showed bigger shifts, while the glass ionomer group “Vitremer” showed stability on its structure. The evaluated mouthwashes displayed similar behavior between each other.

**Conclusions:**

The use of mouthwashes triggered changes on the structure of both dental materials: composite resin and resin modified glass ionomer mostly associated with surface roughness.

** Key words:**Glass ionomer, composite resin, mouthwash, roughness, degradation.

## Introduction

Dental esthetics is the desired result of patients seeking dental treatment. Different restorative direct techniques suggest the use of adhesive composite resin due to its acceptance among the general public and its improved composition, developed in recent years (1). Filler particles, in the case of composite resin (2), have provided the material with exceptional smoothness, brightness, strength, high resistance to abrasion (3), decreased microleakage (4), reduced polymerization contraction and working time (5). In the case of glass ionomers, the addition of methacrylate particles (6) improved the material´s solubility with different chemical substances (7).

Despite all the improvements, the rupture of chemical bonds with the subsequent softening of the material (8) is still a frequent phenomenon especially in unreacted monomers, resulting in an increased solubility of the material, which is defined as sorption (9).This anomaly complements with moisture absorption manifested by the weight gain of the material, which is produced by the infiltration of substances such as saliva, food, liquids and mouthwashes. The moisture penetration produces degradation (10), which is strictly related with the amount of inorganic compound, as well as with its composition, size, volume and degree of conversion (11-13).

Mouth rinses are substances used successfully in the prevention of oral disorders such as caries and periodontal disease (14). Their composition is based on water, antimicrobial agents, salts, preservatives, alcohol and hydrogen peroxide. Mouthwashes trigger a decrease in the oral pH, which has been associated with an increase in sorption and solubility, thus leading to surface degradation and softening of the composite biomaterial (12,15,16).

The constant modifications improving the composition of composite resins and resin modified glass ionomer cements, and their increasing popularity, leads researchers to believe in a possible resistance to this undesired phenomenon. Considering this particular reason, the aim of this study was to evaluate the weight shift and surface roughness in nanohybrid composite resin and resin modified glass ionomer in direct contact with mouth rinses, during different periods of time.

## Material and Methods

An observational experimental study with a convenience sample was proposed, where 144 specimens were manufactured with two resin modified glass ionomer cements (Vitremer 3M-Espe and Lonolux Voco) and two nanohybrid composite resins (Grandio VOCO and Filtek Z250 XT 3M). 36 samples of each material were produced over a nylon matrix (Duralon) with a diameter of 5mm and a thickness of 2 mm. A dental matrix without composite adhesion was used, as well as a dark glass surface to assure smoothness and light refraction.

The resin modified glass ionomer was prepared according to the manufacturer´s instructions, considering the proper dosage, mixing time and insertion. A Centrix syringe was used for the process, waiting the initial setting time of 30 seconds, followed by the material´s polymerization with LED light (Woodpecker B) with a distance of 1 mm for 20 seconds. Composite resin was placed with a direction towards the walls of the matrix, in layers of 2 mm. Each layer was light cured with the same LED light and the same distance and time, as in the resin modified glass ionomer preparation. The samples were polished with Sof-Lex discs, in a decreasing order of granulation.

All the fragments were examined with a magnifying glass to verify the absence of bubbles or any other irregularities. Afterwards, they were placed in sterile boxes with artificial saliva to prevent dehydration, for approximately 12 hours. The samples were later weighted with a precision scale (GM 20) whit a reach of 20 grams to 1 μg and an appreciation of 1 μg.

The surface roughness of all specimens was analyzed in the metrology laboratory with a profilometer (Taylor-Hobson), with a reach of 0.3 to 100 μm (-10 to 300 μin) and an appreciation of 0,003 μm (0,08 μin). Subsequently, they were randomly assigned to one of the three subgroups (n=12) to be tested with the mouthwashes. The mouth rinses utilized for this experiment were: Listerine Zero, Listerine Cool Mint and Listerine Whitening, which were selected because of their high popularity in the Ecuadorian market. All the employed mouthwashes had a similar elaboration and expiration dates. The manufacturer´s recommendations were taken into consideration, concerning the dosage (20 ml) and the time to be employed daily (1 minute).

The fragments were subjected to immersion cycles in the selected mouth rinses and artificial saliva, where each cycle consisted of a complete immersion in a mouthwash for 21 minutes (equivalent to three weeks of use) and afterwards in saliva for 12 hours straight. The sequence was repeated until reaching 546 minutes, corresponding to 18 months of continuous use, where the samples were measured once again for weight and surface roughness (labeled as “time 2”). The cycle was repeated again for an extra 546 minutes, with a total of 1092 minutes equivalent to 36 months of use, and the variables were measured once again (labeled as “time 3”).

The sample´s changes in weight and/or surface roughness through the time periods were considered as main outcomes. A repeated measures analysis of variance (ANOVA) with three factors was performed, where an interrelation between variables was observed with the Greenhouse-Geisser correction test. With a *p* value lower than 0,05, the hypothesis was ruled as statistically significant. All the analysis were conducted in the program IBM SPSS version 22.

## Results

The sample´s data of weight and surface roughness is shown in the  Table 1, at the beginning (Time 1), after 546 minutes (Time 2), and after 1092 minutes (Time 3).

**Table 1:**
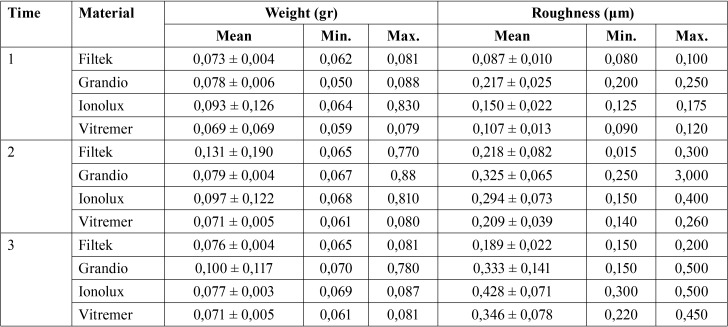
Sample´s description after each period.

The samples, from each assembled material, were randomly assigned to one of the three mouthwashes (cool mint, zero or whitening), and the weight and roughness measurements were recorded afterwards.

**Figure 1:**
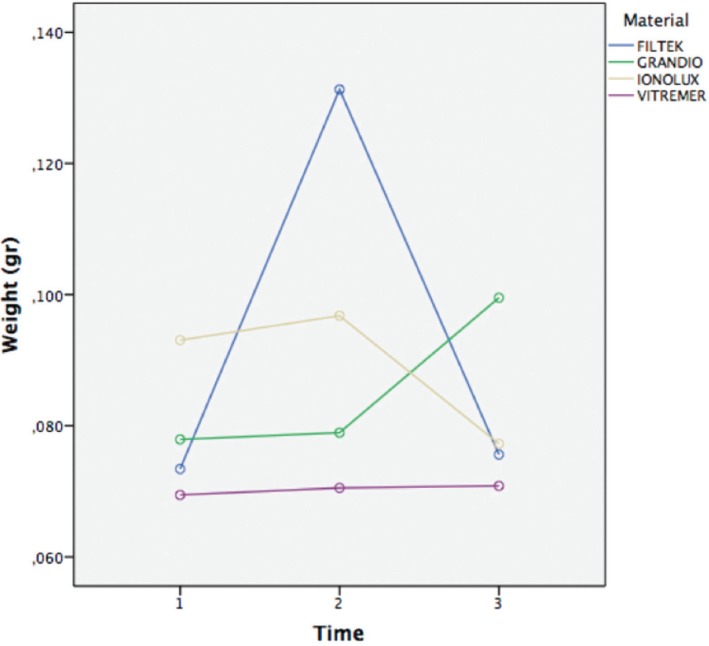
Sample´s weight change over time.

 Table 2 and figure 1 shows data regarding the weight of each material after variance analysis. There was no statistical significance difference when time was evaluated with the material used (*p*=0,08), or when time was compared to the mouthwash utilized (*p*=0,22). However, there was a statistical significance (*p*=0,03) when time was compared to the type of material and mouth rinse used.

**Table 2:**
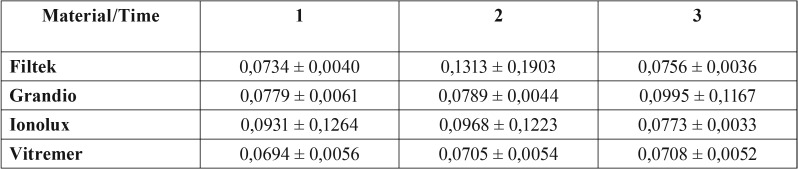
Weight by material and time.

**Figure 2:**
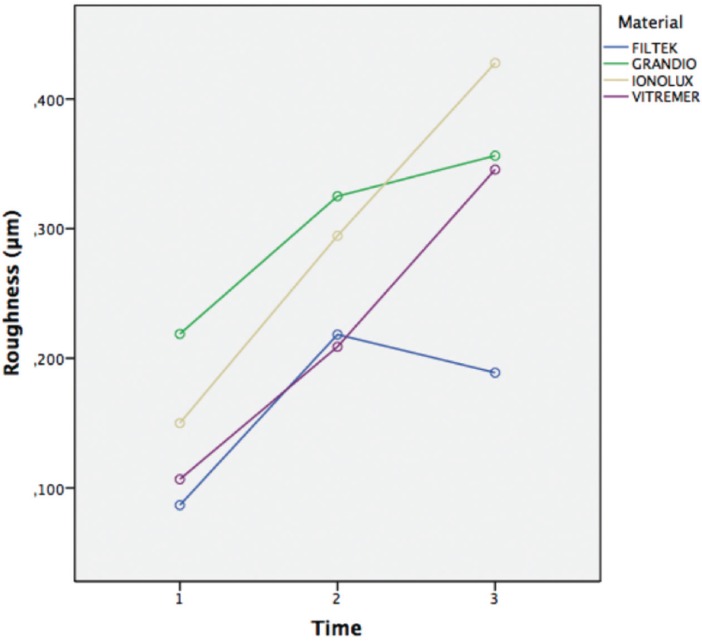
Surface roughness over time.

**Figure 3:**
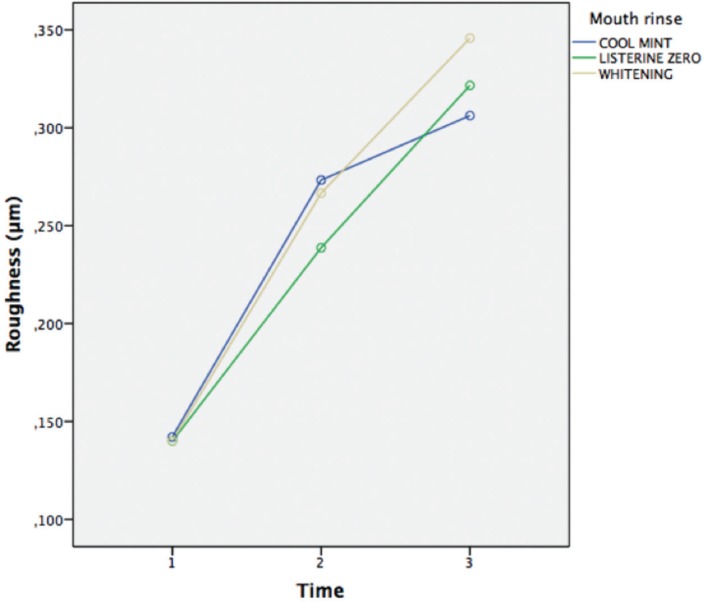
Roughness with each mouth rinse over time.

For roughness, the data are summarized in  Table 3 and  Table 4 as well as in Figures 2 and 3. No statistically significant differences were found when the evolution was compared with the type of mouthwash (*p* = 0,13). On the other hand, when the evolution was compared with the type of sample, as well as the evolution with the type of sample and with the type of mouthwash, statistically significant differences were observed in both cases (*p* <0.001); in most cases the general trend was an increase in roughness.

**Table 3:**
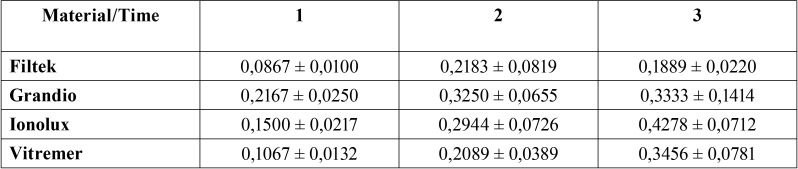
Roughness by type of material and time.

**Table 4:**
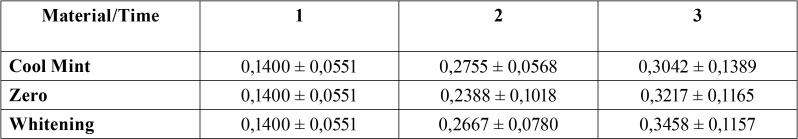
Roughness by mouthwash used and time.

Surface roughness measurements are summarized in  Table 3 and  Table 4 and in Figures 2 and 3. There was no statistically significant difference when time was compared to type of mouthwash (*p*=0,13); nevertheless, when time was compared to the type of material and to the mouth rinses, a statistically significant result was found in both cases (*P*<0,001); in most cases the general trend was an increase in roughness.

## Discussion

The results do not show a difference between the mouth rinses nor the biomaterials (2); however, there is a statistical significance when the two variables cross with each other. This pattern can be explained by the composition of the mouthwashes and the dental restorative materials, where glass ionomer is presented as a material with many clinical benefits (17). The addition of composite elements improves its properties, such as the resistance to hydrolytic degradation, which avoids rupture of chemical bonds and softening through plasticization (4,7,8,18,19).

The study considered two principal outcomes: First, the evaluation of weight shift that showed similar results in all of the samples, independently from the material or mouthwash employed. There was no statistical significance in all the time periods; it can be associated to the composition of the mouth rinses, consisting of hydro alcoholic solutions, vehicle of detergents (12), which affects the hardness and roughness of the materials in a similar way. Nevertheless, even when we didn’t found an overall statistically significant difference, we did found a singularity with one of the composite resins (FILTEK), which had a great change at time 2 and then stabilized at time 3; this kind of behavior can be explained due to the amount of inorganic particles (20) of the material: 63,3% according to the manufacturer, compared with the 89% of inorganic filling of the other composite tested (GRANDIO).

The second outcome considered was the roughness evaluation, which showed a similar behavior of the mouthwashes and a different one compared to the biomaterials, which can be associated to several factors: the formation of cross links in the methacrylate groups of polymers (21), the size of inorganic glass particles and the formation of air bubbles during the material´s preparation (16), which could detach particles, leading to loss of surface integrity and the formation of retention sites, mostly in materials containing glass ionomer in its composition (18,22-25).

Dental professionals frequently look for the most esthetic material with the highest wear resistance properties (26). However, its performance is always dependent on the environment in which they are placed and worked on (1,10). Many substances, including mouthwashes, contact the restorations, influencing on its physical properties due to its composition (9). Since each dental biomaterial has its own advantages and indications, it is mandatory for the professionals to select one based on the environment in which the restoration is going to be placed. In conclusion, the continuous use of mouthwashes affects negatively to the structure of both composite resin materials and glass ionomer ones, mostly on its surface roughness.
